# American Academy of Dental Science

**Published:** 1891-07

**Authors:** 

**Affiliations:** American Academy of Dental Science


### AMERICAN ACADEMY OF DENTAL SCIENCE.

The regular monthly meeting of the American Academy of Dental Science was held in the Boston Medical Library Association rooms on March 4, 1891. President Seabury in the chair.

The paper for the evening was read by Lewis S. Dixon, M.D. Subject, "Proper Care of the Eyesight."

(For Dr. Dixon's paper, see page 423.)

### DISCUSSION.

*Dr. Williams.*—I wish to express my gratification for the privilege of listening to a paper so scientific, and yet so plainly expressed. The subject is one of great importance to us all, and, though I have not made an especial study of it, the principles referred to by the speaker seem to me perfectly sound. The essayist, speaking of rest and intermittent rest, reminds me of a remark that I heard Dr. John C. Warren make when he was professor of anatomy, in 1844, to the class in which I was a student. He said, "When you are doing an operation lasting for half an hour or more, the eyes will sometimes become blurred as a result of close application, and it may be difficult to see without making an extra effort. At such a time you may do harm by forcing the eyes, and it is better to let them rest on some other object for a brief period, or step to the window and look out for a moment or two. In this way the eyes are refreshed, and you can see as well as ever, and by constantly observing this rule there is no danger of exhausting them." The essayist has embodied that principle in his paper, and shown us that rest must alternate with work in order to gain or keep strength. This has proved an invaluable principle to me. Whenever my eyes become tired, not exhausted, I let them rest for a minute on a distant object.

With regard to light reflected into the eyes, we certainly expose ourselves to many disadvantages from light-colored objects and polished metal surfaces. As the essayist says, the light should not enter the eye, but should be a little above and at the back of the operator, in order that he may work with the least effort. Dr. B. S. Codman told me some years ago that he was obliged to give up the practice of dentistry because he found that looking at white teeth seriously affected his eyes. Whether or not that was the first cause, it showed the fact that a constant use of the eyes upon light objects does affect the sight. Some dentists and surgeons wear white operating coats which reflect the light which strikes them, and have about the same effect as a snow-bank under their eyes. Polished metal surfaces and white napkins are likewise to be avoided. We should have the latter shaded a little, so that the light will fall upon the object you are operating on, and not be reflected into the eye.

*Dr. Potter.*—If I understood Dr. Dixon, he recommended the incandescent as superior to any artificial form of light that we have, and I would like to ask him how the incandescent lights can best be prepared,—whether the ordinary fixture, the plain pear-shaped bulb, is proper for either reading or working, or whether that is too bright? It seems to me that there is too much glare, and that the bulb ought to be surrounded with a ground-glass case in order to take off the brilliancy. It has always been my impression that the pear-shaped globe was rather more trying than some gas-lights.

*Dr. Dixon.*—The principal point is to so place the bulb that, as you work or read, you cannot see the filament. If it is above or back of you, or placed in any way so that direct rays from the filament do not strike the eye, then the more light you get, the better. It is the direct light from the illuminated source that annoys.

*Dr. Williams.*—Another point occurred to me, and that is the use of lenses to which the essayist refers. I was taught as a pupil not to imagine that my eyes were microscopes, but to take advantage of the use of the magnifying-glass for looking at small things, just as jewellers, watchmakers, and engravers do, and I always carry a small magnifying-glass with me. I have often operated with a lens on my finger, as an engraver does, and have found its use to be of great service to me. There are aids which civilization affords us, and if we adopt them we certainly have the gain. The young Alpine tourist, in going up the mountain, will always take with him his mountain staff, even if his muscles are all strong and sturdy. That idea of working under magnifying power I once saw put into practical operation by a dentist who used a stand which supported a large, round magnifying-glass, through which he looked into the mouth of the patient and worked as an engraver does. He was formerly an engraver, and after becoming a dentist, and trying for some time to get along without a glass, he was satisfied that he could not do as good work without it. Students should be taught to use a lens, and so improve the quality of their work.

*President Seabury.*—I was advised, when a young man, to use glasses as soon as I found they would help me, and, realizing the necessity of clear sight in my profession, I resolved never to let my sensitiveness override my needs. The person who advised me said, "Don't wait until you are forced to wear glasses, but put them on as soon as they will help your eyes. Then your eyesight will last longer than if you delay till there is an absolute necessity." I have with me to-night my working-glasses, the upper third of which is cut off so that I look through them only when I look down at my work. When I look up I use my natural sight. This arrangement of my glasses involves the principle of alternate rest and use, about which the essayist has spoken, and is very satisfactory to me.

*Dr. Werner.*—The lesson we derive from the essay of this evening is, that we cannot be too careful of our eyesight. To me it is very plain why the average practitioner finds it necessary to use glasses at such an early period of life. Among the causes, as Dr. Williams has suggested, are the bright materials with which we work and are surrounded,—as, for instance, light operating coats, polished and shiny metallic surfaces on our apparatus, the white surfaces of the teeth. These things demand a constant nervous and muscular co-ordination of the eye. Besides, some dentists read at all times and in all lights, even when travelling on railway trains and horse-cars. Every dentist should have a distant outlook from his operating-room, so that the eye may be turned from the near work and rested on a far-away object. We dentists live in small, ill-ventilated operating-rooms for a considerable portion of our lives, and hence it is that so often dentists look pale and haggard, lacking the bright, rosy color of the physically healthy man. Physiologists tell us that the exhalations from the lungs contain a product of decomposition that when reinhaled is very poisonous to all animal life; that it will kill in a very short time, even coming from healthy lungs. How much worse its effect when coming through mouths that are diseased and ill-smelling! We need large operating-rooms with ventilation through a fireplace and windows so as not to live in an atmosphere of exhalations.

*Dr. Taft.*—I should like to ask the essayist if, in his opinion, a fatigued condition of the system brings about a dimness of vision in the hypermetropic eye quicker than in the presbyopic, and in what condition of life dimness would be most apt to manifest itself? I seldom feel any fatigue myself, although I use my eyes very constantly, and work, perhaps, as little with the mirror as almost any man. I know it is not a good thing to do, and have thought that, while feeling but little fatigue, perhaps by the continued strain upon the eyes I may be abusing them.

*Dr. Dixon.*—In simple presbyopia the fatigue is felt only after the age of forty, and it then manifests itself by a dimness of the vision or weariness when engaged in near work. If one does not tax the eyes too much, no serious trouble may be looked for. But in hypermetropia the person is, of necessity, under constant fatigue. As long as his nervous system is able to bear it he may continue using his eyes without noticing any trouble, until some extra demand on, or decrease of, his nervous power makes the defect become prominent. People of sound constitution may have hypermetropia all their lives without inconvenience, and it only comes to their knowledge by having the distant vision fail somewhat when over fifty. All those people who can accept a convex glass and see better with it at a distance have been hypermetropic all their lives; and if they had known it they could have saved their eyes a great deal of unnecessary labor.

In regard to reading in the cars, the only point to remark is this: a normal eye will stand almost any amount of work without becoming fatigued. It is the imperfect eye that gives out. A perfect eye can read in the cars a good deal without serious fatigue or strain; it is able to bear it, though I do not wish to be understood as recommending this practice, for we are not all blessed with perfect eyes, and one might attempt to use an eye with an error so slight that it would be very difficult to find, but which nevertheless would soon give out. As I stated in my paper, the astigmatic eye and the hypermetropic eye never have any rest except when the eyes are closed, and it is the never-ceasing work which they are compelled to do that is at the bottom of the whole trouble.

*Dr. Harris.*—I would make a motion that the thanks of the Academy be extended to Dr. Dixon for the very interesting and valuable paper which he has read before the Academy this evening.

*Dr. Fillebrown.*—I second the motion. I have made no remarks upon the paper, though I have thoroughly appreciated its excellence, and I have learned a great deal more about a pair of eyes that has done good work for a great many years than I ever knew before.

William H. Potter, D.M.D.,
*Editor American Academy of Dental Science.*

